# Mitophagy and mitochondrial dynamics in type 2 diabetes mellitus treatment

**DOI:** 10.18632/aging.203969

**Published:** 2022-03-24

**Authors:** Zhao Shan, Wei Hong Fa, Chen Run Tian, Chen Shi Yuan, Ning Jie

**Affiliations:** 1 Department of Endocrinology, Shenzhen Longhua District Central Hospital, Guangdong Medical University Afliated Longhua Central Hospital, Shenzhen 518110, Guangdong, China

**Keywords:** mitophagy, type 2 diabetes mellitus, autophagy, natural products

## Abstract

The prevalence of type 2 diabetes is associated with inflammatory bowels diseases, nonalcoholic steatohepatitis and even a spectrum of cancer such as colon cancer and liver cancer, resulting in a substantial healthcare burden on our society. Autophagy is a key regulator in metabolic homeostasis such as lipid metabolism, energy management and the balance of cellular mineral substances. Mitophagy is selective autophagy for clearing the damaged mitochondria and dysfunctional mitochondria. A myriad of evidence has demonstrated a major role of mitophagy in the regulation of type 2 diabetes and metabolic homeostasis. It is well established that defective mitophagy has been linked to the development of insulin resistance. Moreover, insulin resistance is further progressed to various diseases such as nephropathy, retinopathy and cardiovascular diseases. Concordantly, restoration of mitophagy will be a reliable and therapeutic target for type 2 diabetes. Recently, various phytochemicals have been proved to prevent dysfunctions of β-cells by mitophagy inductions during diabetes developments. In agreement with the above phenomenon, mitophagy inducers should be warranted as potential and novel therapeutic agents for treating diabetes. This review focuses on the role of mitophagy in type 2 diabetes relevant diseases and the pharmacological basis and therapeutic potential of autophagy regulators in type 2 diabetes.

## INTRODUCTION

Type 2 diabetes mellitus is a prevalent type of diabetes and metabolic disorder disease with the hallmarks of imbalance of energy intakes which trigger metabolic stress as well as inflammatory stimulations, and high levels of fatty acids, triglycerides and LDL cholesterol. These metabolic stress results in dysfunctions of islet beta cell and insulin resistance [1, 2]. Dysfunctions of islet beta cell and insulin resistance predispose to the subsequent stage of disease progression such as cardiovascular disease, neuropathy and nephropathy [3], leading to a huge burden on the healthcare system. Although there are various treatment options (metformin, sulfonylureas, meglitinides, alpha-glucosidase inhibitors) for T2DM, all of them have adverse effects such as metformin triggered intestinal discomfort and reduction of vitamin B12 absorption; sulfonylureas resulted in a loss of efficacy as well as hypoglycaemia [4]. To solve this problem, a novel mechanistic investigation of T2DM pathogenesis is required for developing effective therapeutic approaches. In particular, mitochondrial dynamics plays a pivotal role in the pathophysiology of diabetes. For type 2 diabetic patients have high levels of pyruvate and nonalcoholic steatohepatitis but low levels of ROS productions causing in mitochondrial dysfunctions [5].

Recently, a growing body of evidence supported that alterations of autophagy by autophagy modulators are the potential drugs to deal with diabetes mellitus. Diabetic kidney disease in a murine type 2 diabetes mellitus was alleviated by modulation of autophagy epigenetic regulators(isorhamnetin) via an increase of autophagosomes in renal tissues, then improved blood glucose levels and lipid profiles [6]. Autophagy is a catabolic process to maintain cellular homeostasis by degradation of misfolded proteins and damaged organelles. In general, the unwanted proteins or organelles were sequestered by a double-membrane autophagosome. Afterwards, the cargo contained autophagosome fused with lysosome, which has a variety of degradative enzymes, to digest the cargo and exported it out for recycling [7]. Interference of autophagy functions in pancreatic β-cells, especially the impairment of autophagy flux, has a critical role in the pathogenesis of the development of insulin resistance, hyperinsulinemia and diabetic cardiomyopathy [8–10]. Autophagy is an important catabolic mechanism for pancreatic β-cells homeostasis in terms of cell proliferation, lipid synthesis, lipolysis, glucose uptake as well as responses to stress conditions. Therefore, understanding the underlying mechanism between diabetes development and autophagy pave a new way to design anti-diabetogenic drugs.

Mitophagy is one of selective autophagy that specifically acts on degrading the damaged or dysfunctional mitochondria. Concerning the molecular mechanism, the damaged mitochondria are labelled with the main orchestrators PTEN-induced putative kinase 1 (PINK1), the ubiquitin ligase (PARKIN), ubiquitin and sequestosome-1 (p62/SQSTM1). Afterwards, p62 are conjugated to the autophagosomal microtubule-associated protein 1A1B-light chain3 (LC3), consequently to form an autophagosome capsuled with selected mitochondrial cargos [11]. Emerging evidence has demonstrated that mitophagy is related to the developments in both Type 1 and Type 2 diabetes. It is suggested that mitophagy could be a regulator to remove the excessive ROS during hyperglycaemia conditions [12]. Besides, the reductions of the number of mitophagy autophagosome in diabetic retinas and an increase of oxidative stress in db/db mice through modulation of mitophagy related protein Parkin and PINK1 were observed in the experimental diabetic models [13]. To tackle the impairments of mitophagy flux in diabetes, restoration of mitophagy by mitophagy inducers may be a potential drug target in this aspect.

In an effort to the potential mitophagy inducers in the pathogenesis and progression of diabetes, there are ample studies were performed to examine the therapeutic effectiveness of autophagy modulators in the development of diabetes [14–16]. However, the efficiency of mitophagy inductions of these autophagy modulators is limited. It is required more efforts in exploring the mitophagy induction in this area when the mitochondrial dynamics contributed so much to the pathogenesis and the onsets of type 2 diabetes. Natural products with mitophagy-induction capacities demonstrate as potential candidates in this way. Natural products with mitophagy induction capability plus a wide safety margin and low toxicity profiles are two essential features for designing translational medicine. In order to pave a new way to identify natural compounds for therapeutic intervention for diabetes mellitus, we summarized the recent discoveries of mitophagy inducers with phytochemical characterises such as glucoside, flavonoids and alkaloids and investigate the underlying mechanisms of action involved in protection against diabetes mellitus developments.

## Autophagy related genes and type 2 diabetes mellitus treatment

Autophagy is an evolutionarily conserved homeostatic mechanism. Autophagy is tightly modulated by nutrient conditions, cellular metabolism, energy status and oxidative environments, unwanted protein accumulations. In this section, we briefly point out the major signaling mechanisms relevant to autophagy in diabetes mellitus.

Autophagy related genes (ATG) are essential in regulations of autophagosome initiation, maturation and fusion between autophagosome and lysosome to form autolysosome [17]. There are more than 30 mammalian ATG genes involving in an array of autophagy processes [18, 19]. In the conditions of insufficient nutrients such as glucose and amino acids or specific metabolites. Autophagy is being induced originating from the endoplasmic reticulum. Golgi apparatus, mitochondria as well as plasma membranes [20]. The most canonical pathway involved in the autophagosome initiation process is the mTOR complex 1(mTORC1) dependent signaling pathway. The mTORC1 complex consists of mTORC1, unc-51 like autophagy activating kinase 1 (ULK1) complex, class III phosphor-inositide 3 kinases (PI3K). For instance, phosphatidylinositol 3-kinase catalytic subunit type 3 (VPS34) [21, 22]. Upon the autophagosome expansion, ULK1 phosphorylates Beclin 1 to activate VPS34 and forming phosphatidylinositol-3-phosphate (PI3P). Afterwards, the activated complex and associated proteins (Atg 18, Atg20 and Atg21) are translocated to the phagophore assembly site and then immature autophagosome is conjugated by Atg5, Atg 7, Atg10 and Atg5 for maturation [23–25]. In the end, the mature double-membrane autophagosome is formed and ready to digest the unwanted material [26, 27].

Autophagy related genes have critical implications in insulin deficiency and uncontrolled diabetes. For example, autophagy-related genes Atg5, LC3a and LC3B mRNA in adipose tissues for obesity patients are higher than that of lean patients. Besides, these mRNA levels are associated with insulin resistance and obesity-related metabolic disorder [28]. Besides, murine hepatic samples demonstrated 13 out of 20 autophagy-related genes are downregulated in the context of mice feeding with high fat diets [16].

A recent report demonstrated the linkage between impaired mitochondrial function and metabolic syndrome during T2DM progressions. FUN14 domain-containing 1 (FUNDC1) is a newly identified mitochondrial outer membrane protein as well as LC3B-conjugated protein (mitophagy inducer) to modulate mitochondrial quality control. Mice depleted with FUNDC1 exhibited more severe obesity and insulin resistance due to mitophagy dysfunctions [29, 30].

## Mitophagy controls mitochondrial dynamics

**Figure 1:**
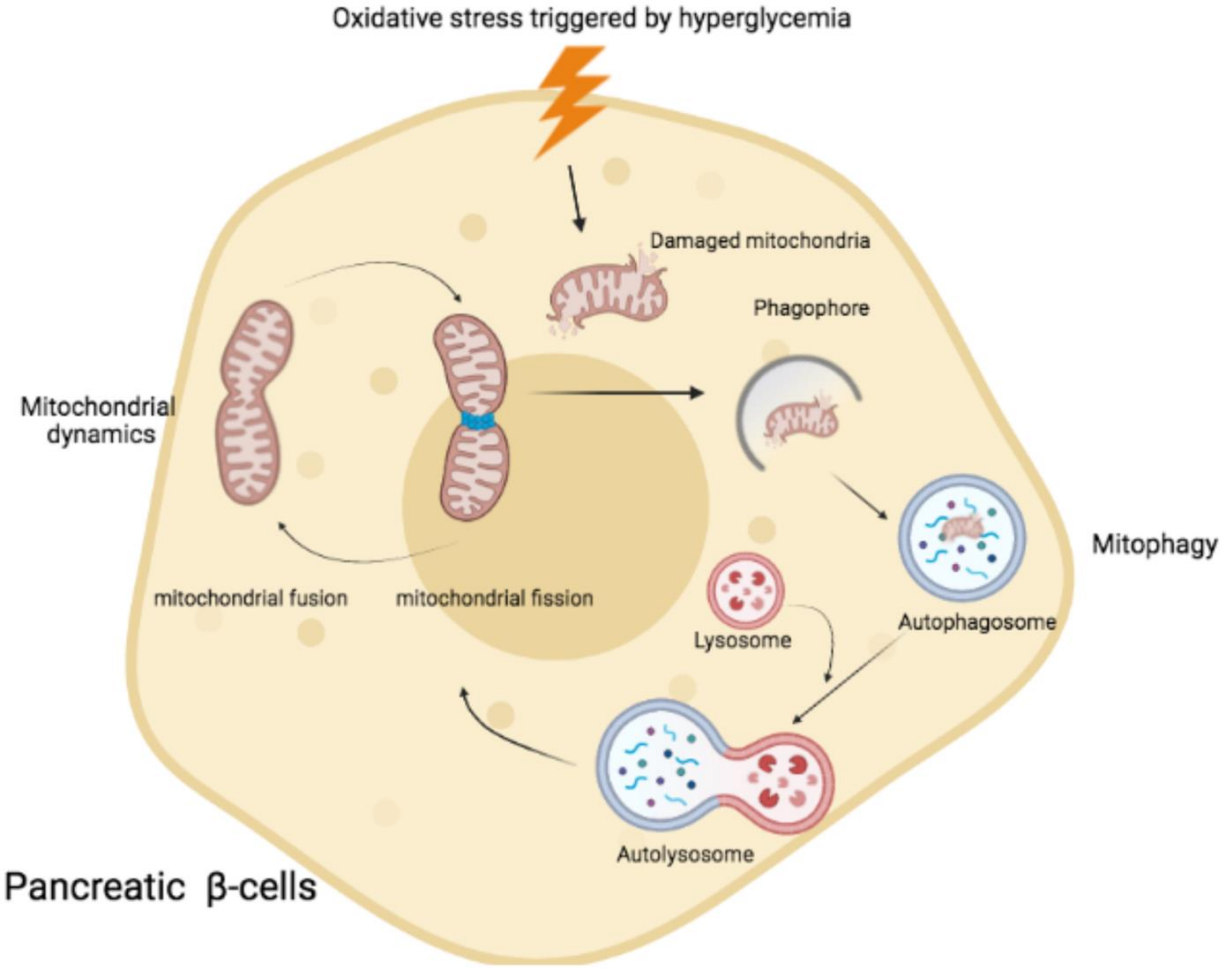
**Schematic representation of mitochondrial fusion and fission process and mitophagy in pancreatic β cells.** Under the condition of hyperglycaemia, oxidative stress triggered the damaged mitochondria. Mitophagy was activated to remove damaged mitochondria by encapsulation of autophagosome. Afterwards, autophagosome fused with lysosome and formed autolysosome to degrade the damaged mitochondria via acidic lysosomal hydrolase.

Mitophagy is a pivotal regulator of mitochondrial homeostasis and effective means for intracellular ROS clearance. A recent study demonstrated that damaged mitochondrion fused with a functional mitochondrion and resulting in a larger damaged mitochondrion and exacerbating the oxidative stress by releasing a high amount of ROS [31]. In the physiological condition of mitochondrial dynamics, mitophagy acts an important role to maintain a healthy mitochondrial population. Mitophagy encapsulated the damaged mitochondrial fragments by tagging them with PINK1 and then phosphorylated PARKIN as well as ubiquitin. Phosphorylated PARKIN further mediated the ubiquitination of the outer membrane for autophagosome formation and fused with lysosome for degradation [32] (Figure 1). The damaged mitochondria were eliminated by mitophagy preceded the stimulations of excessive ROS caused by damaged mitochondria. However, in type 2 diabetes, β-cells are exposed to a high concentration of glucose levels. Mitochondria tend to fission rather than fusion by an increase of Drp1 recruitment and OPA1/MFN degradation as well as mitophagy impairment [33]. The study demonstrated that patients with mild hyperglycaemia compared with the type 2 diabetic patients, type 2 diabetic patients resulted in reductions of mitophagy related genes expressions (NIZ, PINK1, Parkin) [34]. These genes were essential in the mitophagy initiation, and then resulting in the elimination of the damaged mitochondrial fragments failures. More abnormal mitochondria were observed in type 2 diabetic patients with the smaller size of mitochondria [35] as well as swollen and disrupted mitochondria in the hepatocytes from insulin-resistant patients [36]. The accumulation of dysfunctional mitochondria accompanied with aggregated mitochondrial ROS triggered the oxidative damage to the pancreatic beta-cell, eventually facilitating the cell death of beta-cells by apoptosis as well as increasing insulin resistance [32, 37].

## Disorder of mitochondrial dynamics and mitochondrial functions trigger T2DM

Mitochondrial dynamics is controlled by fission and fusion in order to maintain mitochondrial morphology. Mitofusin 1 and 2 are transmembrane proteins that bind and merge adjacent mitochondrial membranes through hydrolysis of guanosine 5’-triphosphate and initiation of fusion process [38]. In T2DM, high glucose levels induced excessive ROS stimulations subsequently enhanced the ratio of Drp1:Mnf2 ratio and lead to mitochondrial fission [39]. The mitochondrial dynamic shifted to the fission and then resulting in aberrant mitochondrial dynamics, insulin-resistance and β-cell dysfunctions [40, 41]. In the same vein, patients with T2D and obesity show decreased levels of MFN2 expression in skeletal muscles that disrupted mitochondrial dynamics and accelerating the development of insulin resistance [42]. Additionally, *in vivo* study, C57BL/6NCrl mice fed with high-fat diet and induction with streptozotocin to develop a DM like mice. The DM like mice demonstrated that increased the protein expression of fission gene (DRP1) and decreased the protein expression of fusion genes MNF2 and OPA1), as well as PPARGc1a (key gene regulator in mitochondrial biogenesis) [43].

Mitochondria regulate insulin secretion through modulations of mitochondrial ATP synthase as well as ATP productions. Mutation of mitochondrial genes m.8561C substitution in MT-ATP6/8 (subunits of mitochondrial ATP synthase) resulted in the failed assembly of mitochondrial ATP synthase and decreasing ATP productions, eventually causing abnormal ROS production and causing cell death by apoptosis [44]. On the other hand, aberrant mitochondria biogenesis caused energy imbalance and accelerated ROS productions, consequently exacerbating pathological pathways of the development of diabetes and associated macrovascular complications (stroke, myocardial ischemia) [45]. Besides, the control of normal mitochondrial fusion and fission by mitophagy is a prerequisite for β-cells survival countering inflammatory stress under hyperglycaemia [46]. Overexpressed the mitophagy regulator gene (CLEC16A) enhanced the damaged mitochondria clearance by activation of mitophagy, restoring the mitochondrial membrane potential as well as pro-inflammatory-triggered cell death of β-cells [32].

Moreover, mitochondria have a self-defence mechanism to counteract overnutrition (high levels of glucose), namely mitochondrial hormesis. When exposing sublethal ROS-induced stress, mitochondria became more resistant to oxidative stress through enhancing the mitochondrial functions by activation of sirtuin 1/3 (SIRT1/3) and AMP-activated protein kinase (AMPK) and PGC-1alpha. This adaptive defence system lead to a decrease in ROS production and ATP synthesis, increasing insulin sensitivity in β –cells [47]. Taken together, it is evident from the above that a strict balance of mitochondrial dynamics (Table 1), maintaining normal mitochondrial functions to remove excess ROS accumulations have beneficial influences on the onset and progression of T2DM.

**Table 1:** Genes involved in mitochondria dynamics.

**Gene**	**Mitochondrial regulation in diabetes**	**Reference**
NRIP1	Reduces respiratory efficiency of mitochondria	[48]
DYRK1A	Inhibition of DYRK1A caused mitochondrial dysfunction	[49]
APP	Activation of APP induced mitochondrial oxidative stress and mitochondrial dysfunction	[50]
RCAN1	Activation of RCAN1 caused mitochondrial dysfunctions	[51]
CBS	Activation of CBS reduced mitochondrial redox activity	[52]
Ndufa4	Inhibition of *Ndufa4* reduced the activation of complex I and IV activity	[53]
SOD1	Inhibition of SOD increased oxidative stress and release of cytochrome C	[54]
ETS2	Activation of mitochondrial death pathway	[55]
PREP1	Inhibition of oxidative phosphorylation and promotion of mitochondrial fusion genes OPA1 and MFN2	[56]

APP, Amyloid Beta Precursor Protein; CBS, Cystathionine Beta-Synthase; DYRK1A, dual-specificity tyrosine- (Y)-phosphorylation regulated kinase 1A; ETS2, ETS Proto-Oncogene 2; Ndufa4, NDUFA4 Mitochondrial Complex Associated); NRIP1, Nuclear receptor interacting protein 1; PREP1, PBX/Knotted 1 Homeobox 1; RCAN1, Regulator Of Calcineurin 1; SOD1, Superoxide Dismutase 1.

## Mitochondrial dynamics is a pivot target for T2DM treatment

Mitochondria dynamics are tightly regulated by mitophagy and also is a major organelle for cellular homeostasis. It is the main energy source for cells and ATP generations. In hyperglycaemia, high levels of glucose are converted to pyruvate and NADH through glucose oxidation in mitochondria. ROS as a by-product is stimulated from mitochondrial complexes I and III [57]. In order to achieve the energetic requirement of cells, mitochondria undergo continuous cycles of mitochondrial fusion and fission to redistribute the populations of mitochondria in the different tissues based on different nutrient conditions [58]. In type 2 diabetes mellitus, the excessive nutrients promoted mitochondrial fission as accompanied by down-regulation of mitochondrial fusion, subsequently resulting in uncoupling respiration. Uncoupling respiration lowered the rate of oxidative phosphorylation and mitochondrial reactive oxygen species productions [59].

Apart from the regulations of mitochondrial fission and fusion switches to regulate mitochondrial dynamics. Morphology of mitochondria also contributed to the progression of T2DM. Compared with a normal person, diabetic patients showed smaller mitochondria in skeletal muscle and low levels of creatine kinase activity [60]. In the condition of hyperglycaemia, more mitochondrial fragmentations have resulted in various types of tissues such as heart, liver and cardiovascular and pancreas [60, 61].

Oxidative stress is an important stimulator in the regulation of β-cells. Dynamin-related protein 1 (Drp1) has a positive correlation with the mitochondrial fusion proteins (mitofusin 1 and mitofusin 2). Dysfunction of Drp1 results in reductions of mitochondrial membrane potential and ATP stimulations, ultimately a decrease of glucose-stimulated insulin secretion [62, 63]. Based on this pathological molecular mechanism, anti-oxidants should be potential candidates for insulin resistance. A recent study demonstrated that SS-31 treated diabetic nephropathy through regulations of mitochondrial membrane potential and ATP modulations as accompanied with suppressing the expression of NADPH oxidase -4 and transforming growth factor-beta 1 (TGF-β1), then further enhancing activation of p38 mitogen-activated protein kinase (p38 MAPK) and NADPH oxidase activity in mesangial cells in the context of hyperglycaemia [64]. Another study also showed that obese patients exhibited lower mitochondrial oxidative characteristics including a decline of mtDNA and mtDNA-mediated translation system in the comparison with their lean co-Twins. This downregulation results in fatty acid oxidation, ketone body production and breakdown, and tricarboxylic acid cycle, eventually causing insulin resistance, adiposity and activation of inflammatory cytokines [65].

## Effects of natural products on T2DM-related tissues through mitophagy

**Figure 2:**
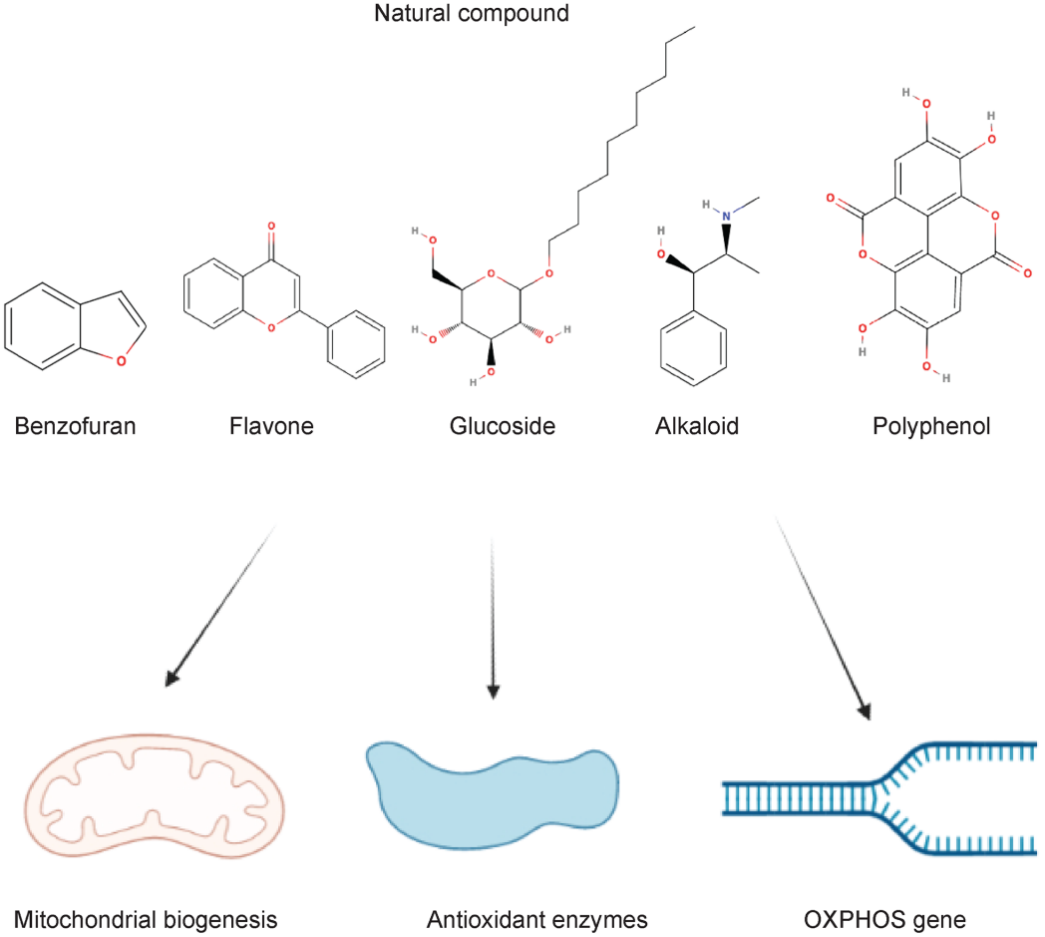
Natural compounds acting on the mitochondrial dynamics in type 2 diabetes and complications.

As mentioned above, mitophagy encapsulated the damaged mitochondria and dysfunctional mitochondria through the autophagy-lysosome pathway for either nutrient requirements or removing excessive ROS from tissues to maintain the mitochondrial dynamics. Under the conditions of over-nutrient (type 2 diabetes), inhibitions of autophagic flux and ROS generations due to impairment of mitochondrial respiration and fragmentation [66, 67]. Natural products modulating mitophagy has been risen concern to improve the T2DM-associated mitochondrial dysfunctions (Figure 2). To assess the therapeutic efficiency of natural products rectifying mitochondrial dynamics through the autophagosome-lysosome axis and potential side effects that have resulted in tissues or cells, T2DM related models and *in vitro* experiments should be performed before translating into clinical applications. The summary in the following sections are including the common use of natural products in treating T2DM *in vitro* and *in vivo* (Table 2).

**Table 2:** Models and signaling pathways of natural products in mitophagy enhancements and mitochondrial dynamics.

**Groups**	**Natural compound**	**Experiment model**	**Signaling pathway**	**Reference**
**N/A**	Ginseng-Sanqi-Chuanxiong	Human aortic endothelial cell	AMPK	[72]
**Benzofuran**	Salvianolic acid B	Human umbilical vein EC line EA.hy926	ROCK1	[73]
**Flavone**	Scutellarin	Human umbilical vein endothelial cells (HUVECs)	PINK1/Parkin	[74]
	Baicalin	Sprague Dawley rats injected with STZ (N=10 per group)	AMPK	[75]
**Alkaloids**	Berberine	H9C2 cells	AMPK	[76]
	Caffeine	C2C12 skeletal myotube	LC3	[77]
**Glucoside**	Salidroside	HT22 cells	mTOR	[78]
	Notoginsenoside R1	Db/db mice, rat retinal Müller cells (rMC-1) (N=12 per group)	PINK1/Parkin	[13]
**Flavonoids**	Kaempferol	intestinal porcine epithelial cells	Nrf2	[79]
	Extracts of bilberry fruits	Male Wistar rats Heart primary culture	N/A	[80]
	Quercetin	C57BL/6J mice fed with ethanol (N= 15 per group)	AMPK ERK2	[81]
**Polyphenols**	Resveratrol	Human umbilical venous endothelial cells	AMPK/HIF1	[82]
	Epigallocatechin-3-Gallate	Goto-Kakizaki (GK) rat	ROS-ERK/JNK-p53	[83]
	Baicalin	Kunming mice Induced with STZ (N= 6-12 per group)	LC3, p62 activation	[84]
**Others**	Trehalose	C17.2 neural stem cells Mice fed with 18% protein and 11% fat diet (N= 4 per group)	LC3	[85]
	Melatonin	SK- N- MC (human neuroblastoma cell line) and SH- SY5Y(human neuroblastoma cell line)	MT2 /Akt/NF- κB	[86]

AMPK, AMP-activated protein kinase; Akt, Protein kinase B; ERK, extracellular regulated protein kinases; HIF1, hypoxia inducible factor-1; JNK, c-Jun N-terminal kinase; LC3, microtubule-associated protein 1A1B-light chain3; MT2, Matriptase-2; mTOR, Mammalian TORC1; NF- κB, nuclear factor kappa-light-chain-enhancer of activated B cells; Nrf2, nuclear factor erythroid 2–related factor 2; Parkin, ubiquitin ligase; PINK1, PTEN-induced putative kinase 1; ROCK1, Rho Associated Coiled-Coil Containing Protein Kinase 1; ROS, Reactive Oxygen Species.

Natural products modulated mitophagy to restore the mitochondrial dynamics in various mechanisms. Salvianolic acid B is a natural class of 1-beznofurans antioxidant that extracted from traditional Chinese Medicine Danshen (Salvia miltiorrhiza). Salvianolic acid B inhibited Rho-associated protein kinase - mediated mitophagy through the reduction of fission protein expressions Dynamin-1- like protein (DRP 1) and mitochondrial fission 1 protein (FIS 1) and subsequent cell death of an endothelial cell in the context of exposure of high glucose conditions and oxidized low-density lipoproteins [68]. Another single compound, namely Ginseng-Sanqi-Chuanxiong (GSC) has protective effects on diabetes-related cardiovascular protections triggered by high glucose and palmitate. Ginseng-Sanqi-Chuanxiong extracts promoted mitophagy to eliminate mitochondrial ROS and autophagosome formation via the activation of AMPK pathway [69].

## Curcumin with mitophagy on T2DM

Curcumin rectified the lipid peroxidation and oxidative stress in several tissues [70]. The antioxidant effects of curcumin are attributed to enhancing mitochondrial biogenesis, reducing the ROS as well as upregulations of antioxidant enzymes. In adipocytes, curcumin increased the mitochondrial biogenesis via activations of AMPK/PGC-1alpha coincident with mitochondria regulator proteins, mitochondrial transcription factor A and nuclear respiratory factor 1 [71]. *In vivo* model of curcumin administration, curcumin exhibited increased mtDNA amounts and increased the protein related to mitochondrial functions such as uncoupling protein 1(UCP1) for proton transfer and ATP synthesis in the matrix of the mitochondrion and the master regulator of mitochondrial biogenesis PGC-1alpha [72]. Besides, curcumin has been reported for mitophagy induction in nasopharyngeal carcinoma CNE2 cells as evidenced by facilitating the swollen mitochondria and mitochondrial membrane impairments [73]. However, the research of curcumin on β-cells have not been reported. More investigation in these areas is warranted. For clinical references, curcumin supplementation (500mg) for 90 days improved the blood glucose as well as body mass index in prediabetes subjects [87]. Furthermore, curcumin intake with 80 mg daily for a total of 12 weeks reduced insulin resistance, LDL-cholesterol and increased total antioxidant capacity as compared with placebo groups [88].

## Caffeine with mitophagy on T2DM

Caffeine is a xanthine alkaloid and major component extracted from green tea, coffee and chocolate. It has been demonstrated to reduce the autophagy-dependent lipid concentration in hepatic tissues through lipophagy [89]. Besides, caffeine also demonstrated that to activate mitophagy for increasing fatty acid oxidation as well as mitochondrial biogenesis as accompanied with potentiated the rate of mitochondrial degradation in skeletal muscle [77].

## Quercetin with mitophagy on T2DM

Quercetin is a key component from Sophora japonica L. and Platycladus orientalis (L.) Fanco. Quercetin has been demonstrated a potential drug for dealing with diabetic neuropathy. Quercetin mitigated ROS generations, ATP synthesis as well as mitochondrial damage in high-glucose -exposed rat Schwann cell line and Streptozotocin (STZ)-induced diabetic rats via promotion of AMPK/PGC-1α pathway [90]. Another study also showed similar a phenomenon on hepatocellular carcinoma cells, quercetin improved the mitochondrial complex I activity and facilitated the binding of PCG-1alpha and PPARα which is responsible for the regulation of energy production and energy utilization in metabolic issues, ultimately potentiated cellular NAD+/NADH and intracellular mitochondrial integrity and redox status of the hepatocytes [91]. Beyond that, quercetin and its derivatives quercetin-3-O-glucuronide have been reported for the amelioration of triglyceride accumulation via fatty acid synthase modulations as coincident with for AMPK activation to restore mitochondrial mass and biogenesis by modulation of mitophagy [92]. In clinical investigations, 500 mg quercetin supplementation daily for 4 weeks decreased plasma uric acid (a risk factor for T2DM) [93].

## Berberine with mitophagy on T2DM

Berberine, a quaternary ammonia compound isolated from Traditional Chinese herbs *Coptis chinensis Franch* and *Phellodendron Chinense Schneid*. Berberine has been reported for treating Alzheimer's disease and dementia through alleviating basal respiration and production of pro-inflammatory cytokines [94]. In diabetes, berberine restored mitochondrial ROS generations, mitochondrial dysfunction and failure of fatty acid oxidation in the models of diabetic kidney disease mouse models. Berberine potentiated the peroxisome proliferator-activated receptor γ coactivator-1α (PGC-1α) signaling pathway, eventually resulting in a balance of mitochondrial energy homeostasis [95]. Apart from diabetic kidney disease, berberine has been demonstrated to ameliorate high glucose-induced mitochondrial fission and fusion disruptions, mitogenesis process. Berberine reduced mitochondria amount as well as cardiomyocyte injury by activation AMPK signaling pathway-dependent mitophagy activation [76]. In clinical approaches, berberine (0.6 g twice daily for 12 weeks)ameliorated the glycated hemoglobin, blood triglycerides, total cholesterol in T2DM patients through regulations of gut microbiome [96, 97].

## Vitamins with mitophagy on T2DM

Given that vitamin B12 deficiency is a common phenomenon in type 2 diabetic patients, vitamin level may a marker for the assessment of type 2 diabetes [98]. Another vitamin, vitamin D3 also reported linking with diabetes. Vitamin D3 administration has been shown to reduce the risk of diabetes [99]. Combination treatment of metformin and vitamin D3 abolished reactive oxygen species and inflammation in diabetes [100]. Besides, another study demonstrated that vitamin D3 also alleviated diabetes-associated complications. High-dose cholecalciferol supplementation of 40,000 IU/week for 24 weeks was associated with improvement in clinical manifestation, cutaneous microcirculation and inflammatory markers in patients with T2DM and peripheral neuropathy [101]. Another clinal trial demonstrated that vitamin D3 supplementation at 4000 IU per day for 24 months did not reduce the risks of diabetes than placebo treatment [102]. The inconsistency may attribute to the dose of vitamins usage in the two experiments. Besides, combined treatment of vitamin and anti-diabetic drugs may result in more benefits than vitamin alone for T2DM treatment. A clinical trial study pointed out that combined therapy vitamin E (400IU twice per day) and pioglitazone 45mg/day reduced the nonalcoholic fatty liver disease activity score in patients with type 2 diabetes mellitus than the patients who only received vitamin E [103].

The above-mentioned compounds have been demonstrated the beneficial effects on the onset and progression of type 2 diabetes mellitus. However, the long-term usage of those natural compounds on type 2 diabetes mellitus may cause potential side effects. Besides, mitochondrial dynamics is a pivot organelle in cellular energy expenditure. Complete inhibition of one part of mitochondrial dynamics will result in deleterious effects on cellular homeostasis. Hence the partial reduction of fission and restoration of mitophagy is the more feasible aim in type 2 diabetes mellitus treatments.

## CONCLUSIONS AND PERSPECTIVES

A growing body of evidence has supported that natural products with autophagy-modifying capacity as potential drugs to treat experimental designed diabetes mellitus. Concerning the underlying molecular mechanism of natural products, they retuned host autophagy (enlarged and inhibited) mainly through AMPK and mTORC1 related pathways to alleviate systemic metabolic disorders.

Although data from *in vitro* and *in vivo* experiments demonstrated natural products elicited therapeutic effects against diabetes mellitus basis on autophagy modulations, clinical trials evaluating the efficiency of natural products as the anti-diabetic drugs are still not well understood. Many questions are warranted before putting on clinical trials. Apart from the clinical limitations, the different experimental parameters researchers used in experiments triggered contradictory results in animal studies. Besides, there is no appropriate tool to measure autophagic flux in human studies. Moreover, natural products usually exhibited multiple metabolic modulations such as enhancing insulin sensitivity, adipogenesis, oxidative stress and mitochondrial integrity at the same time in different tissues types. Tissues-specific approaches to natural products are better to tackle this question.

In this review, we have summarized other aspects of treating diabetes by using natural products in the context of mitophagy regulations. These natural compounds not only regulate the cellular host autophagic mechanisms but also have beneficial effects on host metabolism, inflammatory responses as well as anti-oxidation. A major of them are involved in regulations of AMPK/mTOR pathways to counteract the high glucose level resulting in diabetes. This is expected that these natural compounds may have other features in anti-diabetes aspects. For instance, histone deacetylase (HDAC) inhibitor is a known molecular target in the control of obesity and type 2 diabetes. Baicalein is a flavone from *Scutellaria baicalensis* and *Scutellaria lateriflora* and autophagy inducer with HDAC inhibiting functions, which may contribute to synergistic effects on diabetes [104]. However, this part is still unclear, further studies were warranted for better understanding.

The transcriptional regulation of mitochondrial dynamics and mitophagy also raised concerns. The member of the microphthalmia family of basic helix-loop -helix-leucine-zipper (bHLH-Zip) including transcription factor EB (TFEB), transcription factor E3(TFE3) as well as a microphthalmia-associated transcription factor (MITF) participate in the mitochondrial dynamics in the kinase PINK1 and ubiquitin ligase Parkin -dependent manner [105]. Furthermore, TFEB also controls lysosome biogenesis and coordinating the lysosomal functions [106]. For instance, ATPase pumps (ATP6V1H, Na+/K- ATPase) and lysosomal membrane proteins (LAMP1 and LAMP2) and lysosomal proteases (cathepsin D, cathepsin B). All of them are essential factors to regulate autophagy-lysosome fusion and lysosome acidification [107]. The complete process of mitophagy involved in the autophagosome encapsulates with dysfunctional mitochondria and fuses with lysosome for degradation. Either one of these processes is the foundation for beta cell functions. Research revealed that a lysosomal associated membrane protein-1 (LAMP1) deficient mice impaired autophagic flux. The impaired autophagy flux triggered aggregations of damaged mitochondria and oxidative stress, and eventually causing progressive beta-cell apoptosis and failure [108, 109]. Besides, the regulator of lysosomal genes, TFEB also activated the downstream antioxidant pathway such as nuclear factor E2-related factor 2 (Nrf 2) [110]. A recent study pointed out that degradation of the key suppressor of the antioxidant response, KEAP1 (Kelch-like ECH-associated protein 1) [111] to activate the activity of nuclear factor E2-related factor 2, resulting in increasing transcriptions of anti-oxidant genes and further preventing insulin resistance and increasing insulin production in pancreatic beta-cell, resulting in improvements in metabolic conditions [112].

In addition, several questions should be tackled in the further study of mitophagy. It is well-known that impairment of autophagy caused the failure of damaged mitochondrial clearance and inadequate ROS stimulations contributing to the pathogenesis of T2DM. Therefore, restoring the balance of mitochondrial dynamics and intracellular oxidative conditions are potential approaches for treating T2DM [113].

More importantly, it should be noticed that exercise (improvement in HbA1c, cardiorespiratory fitness and physical features and functional measures in type 2 diabetes patients) increased mitochondrial functions and insulin sensitivity. In detail, exercise improved the mitochondrial functions by restoring the ratio of MFN2/DPR1 in the db/db mice and ameliorated the mitochondrial potential as well as cytochrome C leakage and further prevention of abnormal mitochondrial fission [114, 115]. Apart from exercise, maintaining a healthy diet also demonstrated positive effects in type 2 diabetic patients. A recent research showed that 1.5 months and 3 months of caloric restriction strategy decreased fasting glucose and endogenous glucose production to improving β cell sensitivity in type 2 diabetes and enhancing glucose metabolism respectively [116].

Apart from mitophagy, another selective form of autophagy namely lipophagy has been reported to participate in the pathogenesis of type 2 diabetes mellitus. Lipophagy selectively targeted the lipid droplets for degradations by autolysosome in order to reduce abnormal aggregations of lipid droplets. The reduction of lipid accumulations further prevent insulin resistance and β-cell dysfunctions in the development of type 2 diabetes mellitus [117]. Moreover, same with mitophagy, lipophagy is also activated by exercise and an energy-restrictive diet [118]. The relationship between lipophagy and mitophagy in this regard should take further investigation.

Taken together, mitochondria are dynamic organelles and make great contributions in cellular maintaining homeostasis via regulation of the intracellular ROS and modulation of cell death programs (apoptosis triggered by cytochrome C) [119, 120]. In type 2 diabetes, mitochondrial dynamics and mitochondrial biogenesis, as well as mitophagy were inhibited. Dysregulations of mitochondrial functions and dynamics led to insulin resistance. Rectifying these processes are a potential pharmaceutical candidate for the treatment of these diseases.

To adapt to the metabolic demands of cells, mitochondrial dynamics changed the mitochondrial biogenesis, mitophagy, fission and fusion ratio to match the cellular energy expenditure and nutrient utilization. In an over-nutrient condition such as hyperglycaemia, excess glucose triggered mitochondrial impairment and fragmentation. The fragmented mitochondria inhibited the autophagic flux and facilitating ROS productions. This review summarized the molecular mechanism of mitochondrial dynamics involved in the development of insulin resistance and type 2 diabetes. Besides, we have mentioned natural compounds which enhanced mitophagy and restoration of mitochondrial dynamics. The great benefits from the natural products are fewer side-effects as compared to conventional drugs in an appropriate period [52]. Several natural products have demonstrated antidiabetic potential in animal investigations and clinical trials, this paved the way for them to develop a new anti-diabetic drug. Combination therapy of natural products and conventional drugs will be a novel approach for T2DM. However, there are some limitations on clinical trials such as possible interaction between the conventional drugs and natural products; the stability, bioavailability, therapeutic window and adverse effects of natural compounds in clinical usage are still vague. This way should be investigated in animal studies especially in pharmacokinetics and pharmacodynamics approaches to maximize the utilization of natural products for T2DM management. Therefore, this review may provide a useful summary for helping the researchers to further investigate the novel mitophagy-modulating agents in the direction of treating diabetes in the future.
